# A prospective study of behavioral health indicators and repeat jail admissions among rural inmates

**DOI:** 10.1186/s40352-019-0087-8

**Published:** 2019-04-04

**Authors:** Albert M. Kopak, Kaitlin Guston, Lucas Maness, Norman G. Hoffmann

**Affiliations:** 1 Department of Criminology & Criminal Justice, Western Carolina University, 1 University Drive, Cullowhee, NC 28723 USA; 2 Department of Psychology, Western Carolina University, 1 University Drive, Cullowhee, NC 28723 USA; 3 Evince Clinical Assessments, Inc., 29 Peregrine Place, Waynesville, NC 28786 USA

**Keywords:** Jail inmates, Behavioral health, Mental health, Substance use disorder, Recidivism

## Abstract

**Background:**

Approximately three quarters of a million adults are detained in US jails, and rural detention centers are responsible for the largest recent increases in this population. It is estimated that two thirds of jail inmates meet criteria for a substance use disorder (SUD), nearly half present symptoms consistent with a mental health condition (MHC), and the vast majority of adults in jails have been arrested and booked into these facilities in the past. It is critical to examine the link between SUDs, MHCs, and readmissions to help inform better approaches.

**Methods:**

This prospective study examined the associations between SUDs, MHCs, and jail readmissions in a random sample of 224 adults collected from a rural correctional facility in North Carolina. The Comprehensive Addiction and Psychological Evaluation-5 (CAAPE-5) was administered to participants within 24 to 96 h of admission to the jail. Information consistent with DSM-5 designations for SUDs and several MHCs was evaluated in conjunction with 12-month jail readmission data.

**Results:**

Bivariate analyses demonstrated the disproportionality of SUDs and several MHCs (including depressive episode, posttraumatic stress, and antisocial personality) among adults who were readmitted to the jail. Binary multivariate logistic regression analyses showed SUDs nor MHCs to be associated with any jail readmissions, but multinomial regression results indicated SUDs were the most robust indicator of multiple 12-month jail readmissions.

**Conclusions:**

Local jails need to implement systems capable of conducting behavioral health assessments, with a special focus on SUDs as one of the strongest indicators of readmission. This information will allow jail administrators to better manage detainees while they are incarcerated, but it can also enhance the ability to connect adults with appropriate programming options to address the condition and reduce the likelihood of reentering the detention center.

## Introduction

Jails admit more than 10 million people per year and the vast majority are released back into the community after spending a little more than 2 weeks in the facility, on average (Zheng, 2018). Most of the country’s jail population is located in smaller jurisdictions, and this segment has experienced the largest recent growth in the number of admitted adults (Subramanian et al., 2015). To put this into perspective, nearly 750,000 adults were counted as part of the latest census of jails, which amounts to a jail incarceration rate of 229 per 100,000 (Kaeble & Cowhig, 2018).

Although national estimates of the average number of times adult arrestees have been admitted to local jails remains unknown, information collected from adult male arrestees in several large metropolitan area correctional facilities indicated 80% had been arrested at least once in the past, and from 14% - 29%, depending on the site, had been arrested multiple times in the past year (Office of National Drug Control Policy [ONDCP], 2014). Based on this information, the odds are that most adults who are arrested and processed through the more than 3000 local jails scattered across the country have been admitted at least once in their lives.

Behavioral health conditions must be considered as part of a well-informed approach to the amelioration of multiple jail admissions among adults. Substance use disorders (SUDs) are one of the most pressing concerns observed within this population and a recent report based on the National Inmate Survey indicates 63% of sentenced jail inmates met the Diagnostic and Statistical Manual of Mental Disorders, Fourth Edition (DSM-IV) criteria for drug dependence or abuse (Bronson et al., 2017). A more detailed examination of SUDs among adult jail inmates according to the current DSM-5 criteria has shown the specific types of disorders can vary, but there is considerable evidence to demonstrate alcohol, opioid, and amphetamine use disorders consistently rank among the most prevalent (Proctor et al., 2018; Raggio et al., 2017a, b).

Mental health conditions (MHCs) are also a significant concern among adults admitted to local detention facilities. The best nationwide approximation of the prevalence of MHCs is also gleaned from the National Inmate Survey, which utilized one self-report item to assess whether adults in jail had ever been told by a mental health professional they had any one on a list of 7 conditions (Bronson & Berzofsky, 2017). According to this approach to the assessment of mental health among adults in jails, 44% of inmates were classified with a history of a mental health condition. More detailed work supports these findings with 40–55% of adult jail detainees experiencing the most frequently observed MHCs, which included antisocial personality disorder, posttraumatic stress disorder (PTSD), depression, and manic disorders (Proctor & Hoffmann, 2012; Raggio et al., 2017a, b).

Despite the variation in the assessment of SUDs, and the lack of detail involved in identifying MHCs among large multi-site samples of adults in local jails, it can be accurately concluded that a large proportion of this population suffers from SUDs, MHCs, or a combination of both. Coupled with the incarceration history of many adults in local correctional facilities, it is likely that these conditions significantly contribute to jail admissions. There is scant evidence to support this assertion, but most of the work in this area has utilized disparate behavioral health indicators while focusing on an equally large variety of measures that are usually referred to as recidivism. For instance, adults who had been previously convicted of any offense and who received a SUD diagnosis (according to the International Classification of Diseases, Ninth revision [ICD-9] criteria) by a physician were significantly more likely to be reconvicted of a new offense compared to adults who did not receive a similar designation (Rezansoff et al., 2013). This finding converges with results observed in Philadelphia County, Pennsylvania which demonstrated adults who were formally diagnosed with a SUD (defined according to Medicaid claim designations for DSM-IV abuse or dependence criteria) were significantly more likely to return to the jail over a four-year period compared to adults with no diagnosis (Wilson et al., 2011). This group of adults was also found to return to jail in a shorter period of time compared to those who did not meet similar SUD criteria, potentially putting them at greater risk for being cycled through the criminal justice system with much greater frequency (Wilson et al., 2014).

These studies indicate adults with a SUD designation tend to be more likely to be admitted to local jails, but the important limitations that prevent the ability to make broad generalizations are readily apparent. One of the most obvious is the utilization of formal healthcare records, which glosses over the reality that many adults with similar conditions do not receive formal diagnoses during the average-length jail stay. Another is related to various measures of recidivism, which vary from rearrest to reconviction and sometimes includes self-reported re-offense. Jail admissions occur following an arrest, but not all of them result in reconviction, making this an irrelevant measurement for local detention centers who are most interested in efficiently managing the flow of detainees who pass through their facilities. Additional work is required to examine the relationship between SUDs and jail readmissions with more consistent measures that are not restricted to physicians’ diagnoses, records of treatment engagement that infer the presence of a SUD, or measures of reconviction that fail to clearly capture indicators of readmission into local facilities.

Research on the relationship between MHCs and repeat incarcerations has also identified an increased likelihood that adults who present symptoms of mental illness are more likely to enter detention centers, remain imprisoned for longer periods of time, and return to these facilities quicker than adults without similar conditions. To illustrate this point, evaluation work with specialty programs designed for adults in jail with MHCs has shown more than 40% were rearrested within one year of release with many arrested multiple times and spending several months in local jails (Castillo & Alarid, 2011; Constantine et al., 2010).

Despite the seemingly evident connections between SUDs, MHCs, and reincarceration, significant gaps remain in our knowledge about how these behavioral health indicators are related to readmission to local detention centers. Most of the work in this area has been conducted with prison inmates, which consists of an important population to study, but SUDs and MHCs have been shown to be more prevalent among adults in jail relative to prisoners (Bronson & Berzofsky, 2017; Bronson et al., 2017). In addition, jail administrators are well aware of the return rates of inmates with serious mental illness (AbuDagga et al., 2016), but the inherent challenges in working this population have left most researchers relying on state databases from large metropolitan areas which contain outdated formal diagnostic classifications. There have been significant changes to the classification systems for SUDs and MHCs with the advent of the DSM-5 (Kopak et al., 2014a; Kopak et al., 2014b), leaving a noticeable gap in the knowledge related specifically to the presentation of these conditions among adults in local jails. Most local detention centers are also situated in semi-urban or rural areas, and they tend to lack the highly trained clinical staff who are capable of performing these diagnostic interviews (Applegate & Sitren, 2008). This raises the question of whether the results observed in large urban jails are representative of the patterns emerging from facilities in small jurisdictions. Jails also serve as key sites for this work because these facilities can serve as de facto community behavioral health hubs based on the fact they are the first point-of-contact for adults as they are processed into all other branches of the criminal justice system.

Given the existing limitations in the current knowledge in this area, along with the potential for SUDs and MHCs to serve as significant contributors to reincarceration, the current study was designed to examine the specific relationships between these behavioral health indicators and jail readmissions among adults in a rural area. Specifically, the two primary objectives were: 1) to evaluate the extent to which SUDs and MHCs were associated with jail readmissions among adult detainees, and 2) to determine whether SUDs were more predictive of jail readmissions compared to MHCs. Considering the acknowledgements made by jail administrators that inmates with behavioral health needs seem to appear more frequently in their facilities, and the potential to address these needs with appropriate alternatives to jail detention, the identification of specific indicators of this increased likelihood of being booked into the jail represents an incremental step toward the amelioration of these problems.

## Methods

This study was part of a larger effort to examine the behavioral health needs among adult jail detainees in a semi-rural community in Western North Carolina. According to the US Census classification criteria, the county serving as the study site is classified as an urban cluster adjacent to a county representing an urban area (United States Census Bureau, 2019). During the collection period, which was initiated in December 2015 and concluded in August 2016, the county serving as the study site had a population of nearly 60,000. Recent Census estimates indicated the median age in this area was 47 years, 46% of the population was male, and the vast majority (96%) of the population’s racial and ethnic background was recorded as White (United States Census Bureau, 2018). In terms of socioeconomic markers, 44% of the population in this area completed some college or earned an associate’s degree, and the median annual income among residents over 25 years of age was $31,500.

The facility where this study was conducted has many characteristics typical of a small-to-moderately sized rural jail. The detention center is designed explicitly for adult pretrial detainees, as well as inmates who have been sentenced to remain in custody for a period lasting no longer than one year. The jail operates near capacity with a daily census count ranging from 100 to 125, which is similar in size to approximately one quarter of the jails in the country (Zheng, 2018). As demonstrated in previous research examining the differences between rural and urban jails, this facility does not have full time medical personnel (Applegate & Sitren, 2008). This leaves the jail unable to perform routine comprehensive behavioral health work-ups, but the facility does employ one healthcare professional on a part-time basis to conduct triage assessment.

Adults who were booked into the jail within the previous 24 to 96 h were eligible to participate in the study with the goal of inviting all eligible detainees prior to their first appearance in front of a judge, which was their first opportunity to be released from the facility. All recently booked detainees were eligible to participate, regardless of county resident status. The selection process involved randomly drawing prospective participants’ names from a cup. After inviting selected detainees to participate, they were informed their responses would remain confidential and any information provided could not influence their current legal status. Detainees who agreed to participate signed consent forms approved by the Institutional Review Board of the university with which the researchers are affiliated. Detainees did not receive any incentives for their participation in the study.

A member of the research team conducted a clinical interview with each participant to collect information related to a range of behavioral health conditions. These structured assessments do not require a formal certification, but the research staff member who conducted them was formally trained and supervised in their administration by the instrument’s author. The interview consisted of the Comprehensive Addiction and Psychological Evaluation-5 (CAAPE-5; Hoffmann, 2013), which was designed to identify many behavioral health issues in a manner that is consistent with the fifth edition of the Diagnostic and Statistical Manual of Mental Disorders (American Psychiatric Association, 2013). This instrument has been validated for use with various correctional samples in prior work (Proctor et al., 2014; Tracy & Carkin, 2016; Kopak et al., 2014a, b). Although the information gathered with the CAAPE–5 is used to detect probable diagnoses for research purposes, it is recommended that formal diagnoses be confirmed by a licensed professional for routine clinical use. The CAAPE-5 also gathers information related to demographic background characteristics while simultaneously providing an assessment of common mental health conditions and disorders for many substances (i.e. alcohol, marijuana, cocaine, heroin, amphetamines, hallucinogens, and inhalants). The complete interview can take from 20 to 35 min, depending on the number of substances used and key mental health items endorsed by a participant. A total of 283 interviews were completed as part of the project.

In addition to data collected from the behavioral health assessment interview, booking information was extracted from the jail’s records management system. These data included the types of offenses for which detainees were admitted into the jail, the severity (i.e. misdemeanor or felony) of these charges, and a retrospective measure indicating whether or not the inmate had been admitted to the jail serving as the study site in the 12-month period immediately preceding the interview. Initial assessment of these jail records indicated 24% (*n* = 67) of the 283 participants had been admitted into the jail one time in the past and 43% (*n* = 122) had been admitted on multiple occasions.

After these data were extracted from the jail records management system, the researchers allowed 12 months to lapse. In September 2017, a member of the research team returned to the correctional facility to extract admission data pertaining to the 12-month period between the initial clinical interview and the current data collection. Similar information was collected during this round of data extraction, including offense types, severity of charges, and subsequent jail admissions. For those who were readmitted to the jail, the researchers were able to calculate the number of days detainees were held in the facility. This indicator served as a measure of time-at-risk for readmission and ranged from a minimum of one day to a maximum of 218 days with detainees spending an average of 24.2 days (*SD* = 35.3) in the detention facility. Several inmates were also transferred to various state department of corrections (DOC) facilities to serve a sentence after their clinical interview had taken place in the jail, which meant that follow-up data could not be obtained for these inmates. Among the 283 detainees with whom interviews were conducted, 21% (*n* = 59) were transferred into the custody of the DOC rendering them ineligible for the current study because they did not have the possibility of release or readmission to the jail. In other words, 79% of the total sample was sentenced to remain in custody of the jail, which maintained their eligibility by allowing for their release, and corresponding risk for readmission, within the one-year study period.

A preliminary comparison of the demographic background characteristics of inmates who were transferred to the DOC and those who were eligible for the current study demonstrated more similarities than differences. Transferred inmates were similar in age (*t*(281) = 0.54, *p* = .059), race or ethnicity (χ^2^(1) = 2.84, *p =* .092, Cramér’s = .10), marital status (χ^2^(1) < 0.01, *p =* .956, Cramér’s < .01), and educational attainment (χ^2^(1) = 1.36, *p =* .244, Cramér’s = .07). There was only one difference between these two groups. Nearly nine out of ten inmates (88%) who were transferred to the custody of the DOC were male (χ^2^(1) = 10.97, *p =* .001, Cramér’s = .20) compared to two thirds (66%) of those who were not transferred.

The final study sample of adult detainees who remained in the local jail consisted of 224 participants with a mean age of 33 (*SD* = 10.4) years. Regarding demographic background factors, there were more male (66%, *n* = 148) detainees compared to female detainees and most (60%, *n =* 135) reported not working during the period immediately preceding their admission to the jail. More than two thirds (67%, *n* = 151) of detainees indicated they had completed high school or had some formal education beyond high school. Half (50%, *n =* 113) of the sample of detainees reported they were single and had never been married in the past.

The analytical approach in the current study was conducted in three phases. First, prevalence rates of specific mental health and substance use disorders were examined to describe the extent of behavioral health conditions in the sample of adult jail detainees. Second, a series of chi-square tests of independence were conducted to assess associations between these behavioral health indicators and jail admissions after the clinical interview. Third, a series of multivariate logistic regression models was estimated to examine the associations between behavioral health indicators and jail admissions while controlling for potential confounding variables (e.g. demographic background factors, previous jail admissions, and length of detention as a measure of time-at-risk for readmission). This series of multivariate regression models included a binary logistic model to examine a dichotomous indicator of whether detainees were admitted into the jail on any subsequent occasions, as well as a multinomial logistic model to examine the relative risk of being admitted into the jail on multiple occasions as it related to certain behavioral health indicators.

## Results

Behavioral health conditions were prevalent in this random sample of adult jail detainees. Beginning with substance use disorders, opiate use disorder (primarily heroin) and amphetamine use disorder (primarily methamphetamine) were the most prevalent in the sample. This was likely a reflection of the high rates of opiate use recorded as part of the nation’s unprecedented epidemic, which have been documented among formerly incarcerated adults during this period (Ranapurwala et al., 2018). Approximately one third (34%, *n* = 76) of detainees met criteria for a moderate or severe opiate use disorder and 43% (*n* = 97) met criteria for a moderate-to-severe amphetamine use disorder. Further investigation demonstrated that 30% (*n* = 51) of the 173 detainees who met criteria for one or the other of these two substance use disorders actually met criteria for both, making it difficult to examine the individual impact of either one of these conditions in isolation of the other. There are also unique challenges associated with the treatment of opiate use disorder in combination with other SUDs that are less likely to be observed in cases with single conditions or those involving other substances (Schuckit, 2016). Thus, the high prevalence of this combination of substance use disorders in the sample, the unique difficulties associated with their treatment, and the increased risk for overdose among recently released jail inmates (Lim et al., 2012) served as the basis for examining detainees who met criteria for these substance use disorders relative to those who did not meet similar criteria throughout the analyses conducted in the current study.

There was also a high prevalence of MHCs in the sample. Approximately eight out of ten (79%, *n* = 178) detainees reported symptoms consistent with major depression, a manic episode, PTSD, panic disorder, or antisocial personality disorder. In terms of each condition, 53% (*n* = 119) of the sample met criteria for a major depressive episode, 33% (*n =* 73) reported symptoms of a manic episode, half (50%, *n* = 113) presented symptoms of PTSD, 30% (*n* = 68) met criteria for panic disorder, and 45% (*n* = 100) met criteria for antisocial personality disorder. Although recent national estimates on all of these specific mental health conditions are not available for the population of jail inmates in the US, these data are consistent with recent reports identifying major depressive disorder as the most prevalent of the ones that have been observed (Bronson & Berzofsky, 2017).

Initial examination of jail readmission rates demonstrated almost half (49%, *n* = 109) of the sample was readmitted during the follow-up period. Among adults in the study sample who were readmitted, slightly less than half (46%, *n* = 50) were readmitted on one occasion with the remainder (54%, *n* = 59) experiencing multiple readmissions. Adults who were readmitted several times were booked into the detention facility between three and four times, on average (*M* = 3.5, *SD* = 2.0). Because adults who are readmitted into a local detention facility are likely to draw more resources as they are processed in-and-out of the jail, and those who are readmitted multiple times demanding the most attention, three categories were created to assess the influence of SUDs and MHCs on the likelihood of 1) any readmission, 2) one readmission, and 3) multiple readmissions. This approach also allowed for the ability to determine whether SUDs and MHCs contributed independently to an increased risk for multiple readmissions to the jail relative to zero or one readmission.

Prevalence rates of SUDs and MHCs were examined according to jail admissions and these results are presented in Table 1. Comparisons of detainees who met criteria for SUDs or MHCs relative to those who did not present indications of these conditions revealed several significant differences between these groups. For instance, a significantly larger proportion of detainees who met criteria for moderate-to-severe opioid or amphetamine use disorder were readmitted to the jail (55%) compared to those who did not (42%) meet similar criteria (χ^2^(1) = 4.20, *p =* .040, Cramér’s = .14). This trend was supported with a larger proportion of detainees who met criteria for these SUDs becoming readmitted to the detention facility on multiple occasions compared to those who did not meet similar criteria (χ^2^(2) = 6.28, *p =* .043, Cramér’s = .17). Beyond the statistical importance of these results, it is worth reiterating the fact that over half of detainees with moderate-to-severe opioid or amphetamine use disorders were readmitted at any point during the study period and one-third of detainees who met these criteria were readmitted to the detention facility on multiple occasions making this a significant cause for concern among all adults who entered the jail.

**Table 1:** Prevalence rates for select mental health conditions by 12-month jail admission

Condition	Any readmission	Multiple readmissions
	Prevalence (%)	Prevalence (%)
Substance use disorder*		
Condition	55	33
No condition	42	19
Major depressive episode*		
Condition	57	31
No condition	39	21
Manic episode		
Condition	51	27
No condition	48	26
PTSD		
Condition	55	32
No condition	42	21
Panic disorder		
Condition	54	32
No condition	46	24
Antisocial Personality*		
Condition	60	34
No condition	40	20

Substance use disorders include moderate-to-severe opiate and amphetamine cases. *Differences between readmission groups were significant, *p* < .05

Mental health conditions were also prevalent among adult jail detainees who were readmitted to the jail, which are presented in Table 1 as well. Across the board, adults with MHCs were more likely to be readmitted to the jail relative to those who did not present criteria consistent with these conditions, but not all of these differences were statistically significant. A larger proportion of detainees who reported symptoms consistent with a major depressive episode (57%) were readmitted compared to detainees who did not (39%) report similar symptoms (χ^2^(1) = 7.31, *p =* .007, Cramér’s = .18), but detainees with indications of a manic episode (χ^2^(1) = 0.18, *p =* .673, Cramér’s = .03) or panic disorder (χ^2^(1) = 1.29, *p =* .256, Cramér’s = .08) were no more likely to be readmitted relative to detainees who did not present indications of these conditions. Detainees who reported symptoms consistent with PTSD also experienced similar rates of readmission compared to those who did not present indications of this condition (χ^2^(1) = 3.52, *p =* .061, Cramér’s = .13). In contrast, a significantly larger proportion of detainees who endorsed items consistent with antisocial personality disorder experienced any readmission to the jail (χ^2^(1) = 9.30, *p =* .002, Cramér’s = .20), as well as multiple readmissions (χ^2^(1) = 9.64, *p =* .008, Cramér’s = .21) during the study period.

A binary logistic regression model was estimated to assess the associations between behavioral health indicators and jail readmission while controlling for demographic background characteristics and potential confounding variables, such as previous jail admissions and amount of time detainees were detained. The results from this analysis are presented in Table 2. There was one significant association observed in this model. Detainees who were held in the jail at any point in the 12-month period prior to the initial clinical interview were 3.5 times (*OR =* 3.53, 95% *CI =* 1.85–6.72) more likely to be readmitted to the jail in the 12-month period following the clinical interview compared to detainees who were not previously admitted to the jail.

**Table 2:** Multivariate logistic regression results predicting any 12-month jail readmission

Variable	*β*(SE)	Wald’s χ^2^	*p*	*OR*	95% CI	
					Lower	Upper
Age	.04(.39)	0.01	.922	1.04	.49	2.21
Female	−.64(.35)	3.33	.068	.53	.27	1.05
Non-white	−.67(.41)	2.60	.107	.51	.23	1.15
Unemployed	.16(.32)	0.26	.609	1.18	.63	2.21
Never married	.10(.34)	0.09	.766	1.11	.57	2.16
Less than HS education	−.02(.33)	0.00	.952	.98	.51	1.88
Prior jail admission	1.26(.33)	14.67	.000	3.53	1.85	6.72
Length of incarceration	−.01(.00)	3.66	.056	.99	.98	1.00
Manic episode	−.33(.36)	0.83	.361	.72	.35	1.46
Depressive episode	.62(.34)	3.18	.074	1.85	.94	3.64
Antisocial personality	.60(.31)	3.66	.056	1.82	.99	3.38
PTSD	.41(.36)	1.31	.253	1.51	.75	3.04
Panic disorder	−.29(.37)	0.63	.427	.75	.36	1.54
Substance use disorder	.59(.32)	3.35	.067	1.80	.96	3.36

A multinomial regression model was estimated to further examine the relationship between behavioral health indicators and jail readmissions. This model treated jail readmissions as a tripartite variable designating adults who were not readmitted to the jail in the 12-month follow-up period as the reference group, a second group, which was readmitted to the jail on one occasion, and a third group, which was readmitted to the jail on multiple occasions. These results are presented in Table 3. Similar to the previous multivariate model, detainees’ age, gender, racial and ethnic background, employment status immediately preceding admission, marital status, education level, and length of prior incarceration were all included as control variables. In terms of behavioral health items, there was one significant indicator of increased risk in the comparison between detainees who were readmitted on one occasion relative to detainees who were not readmitted to the jail. After controlling for length of incarceration, detainees who were admitted to the jail prior to the initial clinical interview were more than 4.5 times as likely to be readmitted to the jail compared to detainees who were not admitted prior to the clinical interview. The confidence interval for the estimate of this association ranged from 1.93 to 10.82, which is indicative of a large standard error. Further diagnostics were conducted and VIF values ranged from 1.03–1.32 with a mean of 1.18 indicating sufficiently low levels of multicollinearity between the independent variables in the model. Thus, the relatively large interval is likely due to the restricted numbers of detainees who fell into different readmission groups and should be interpreted accordingly.

**Table 3:** Multinomial regression results predicting 12-month jail readmissions

Variable	One readmission vs. no admissions			Multiple readmissions vs. no admissions		
	Coefficient (se)	Relative risk ratio	95% C.I.	Coefficient (se)	Relative risk ratio	95% C.I.
Age	−.42(.50)	.66	.25–1.75	.38(.45)	1.47	.61–3.51
Female	−.03(.43)	.97	.42–2.24	−1.17(.44)	.31**	.13–.73
Non-white	−.67(.53)	.51	.18–1.46	−.69(.41)	.50	.19–1.33
Unemployed	−.13(.39)	.88	.41–1.81	.39(.39)	1.47	.69–3.16
Never married	.14(.42)	1.15	.51–2.61	.08(.41)	1.08	.49–2.41
Less than HS education	.01(.41)	1.01	.45–2.27	−.06(.39)	.94	.43–2.04
Prior jail admission	1.52(.44)	4.56**	1.93–10.82	1.05(.31)	2.87**	1.32–6.24
Length of incarceration	−.00(.00)	.91	.99–1.01	−.01(.01)	.99*	.97–.99
Manic episode	−.17(.44)	.84	.36–2.01	−.41(.43)	.61	.26–1.41
Depressive episode	.62(.43)	1.85	.80–4.28	.61(.42)	1.84	.82–4.16
Antisocial personality	.59(.39)	1.81	.85–3.86	.51(.38)	1.82	.87–3.81
PTSD	.13(.44)	1.14	.48–2.71	.67(.43)	1.95	.84–4.52
Panic disorder	−.39(.45)	.67	.28–1.64	−.21(.43)	.81	.35–1.88
Substance use disorder	.20(.39)	1.22	.57–2.63	.93(.39)	2.53*	1.18–5.40

*P* values: **p < .01; *p < .05

There were four significant indicators of increased risk observed in the comparison between detainees who were readmitted to the jail on multiple occasions compared to detainees who were not readmitted to the jail in the 12-month period following the initial clinical interview. Female detainees experienced significantly lower risk of multiple readmissions (*RRR =* 0.31, *95% CI =* 0.13–0.73) to the jail relative to male detainees. Detainees who were admitted to the jail prior to the initial clinical interview were 2.8 times as likely to be readmitted (*RRR* = 2.87, *95% CI =* 1.32–6.24) compared to detainees whose first admission took place at the time of the clinical interview. Additionally, lengthier periods of incarceration were associated with lower risk for readmission to the jail (*RRR* = 0.99, *95% CI =* 0.97–0.99), presumably due in part to the fact that adults had fewer opportunities to be readmitted while spending longer periods of time in detention. Finally, detainees who met criteria for moderate-to-severe opiate or amphetamine use disorder were more than 2.5 times as likely to be readmitted to the jail (*RRR* = 2.53, *95% CI =* 1.18–5.40) compared to detainees who did not meet criteria for these substance use disorders. Additional analyses were conducted to examine whether detainees with amphetamine use disorder only, opiate use disorder only, or both were more likely to be readmitted to the jail on multiple occasions relative to those who did not meet diagnostic criteria for these disorders. These results indicated those with opiate use disorder only were over three times as likely (*RRR* = 3.74, *95% CI =* 1.19–11.77) to be readmitted on multiple occasions while those with amphetamine use disorder were also significantly more likely (*RRR* = 3.89, *95% CI =* 1.56–9.69) to be readmitted on multiple occasions compared to detainees who did not meet criteria for one of these substance use disorders.

Post-hoc analyses were also conducted to evaluate potential differences between detainees who were readmitted to the jail on multiple occasions and those who were readmitted once during the 12-month follow-up period. This multinomial logistic regression model treated detainees who were readmitted once as the reference group and there was one significant result. Female detainees were significantly less likely to be readmitted on multiple occasions relative to male detainees (*RRR* = 0.31, *95% CI =* 0.12–0.84). Although none of the behavioral health indicators was significantly associated with multiple jail readmissions in this model, it is worth noting that detainees who met moderate-to-severe opiate or amphetamine use disorder were more than two times as likely as those who did not meet similar criteria to have multiple jail readmissions versus one readmission (*RRR* = 2.07, *95% CI =* 0.88–4.88, *p* = .097).

A final post-hoc analysis was conducted to examine differences in the probability of jail readmission among detainees who met criteria for either a) multiple mental health conditions, b) multiple substance use disorders, or c) any mental health condition plus a substance use disorder. One significant association was observed with detainees who met criteria for multiple substance use disorders and multiple mental health conditions. This group was nearly three times more likely (*RRR* = 2.89, *95% CI =* 1.32–6.35, *p* = .008) to be readmitted to the jail on multiple occasions relative to those who were not classified with similar combinations of conditions.

## Discussion

This study was designed to assess the extent to which SUDs and MHCs were associated with readmissions into a rural county detention center of a similar size and location as many jails across the country, which are also likely to lack full-time behavioral health clinicians. Results demonstrated the high rates of various conditions in the sample, and some of these were indeed related to an increased likelihood of jail readmission. In terms of MHCs, symptoms consistent with major depression, PTSD, and antisocial personality disorder were disproportionately observed among adults who were readmitted to the jail, confirming the reports of jail administrators who have reported that readmitted adults demonstrate greater mental health needs compared to those who are not readmitted (AbuDagga et al., 2016). These results coincide with prior research conducted with adults in the San Francisco jail system, which revealed those with PTSD were significantly more likely to be rearrested compared to adults without similar symptoms (Sadeh & McNeil, 2015). In addition, antisocial personality scales have been found to have some of the largest effect sizes in the prediction of various measures of reoffense, emphasizing the need to indentify adults who may present symptoms consistent with this condition (Katsiyannis et al., 2018). Conversely, the lower rates of panic disorder and reports of experiencing a manic episode were not associated with the likelihood of jail readmission. It is possible that the effects of these specific conditions do not explicitly contribute to behaviors that are likely to result in arrest and jail readmission, but additional work is necessary to investigate these mechanisms, especially among adults booked into local detention centers.

The most noteworthy finding of the current study was related to the association between moderate-to-severe SUDs and multiple jail readmissions. Although this relationship has not been examined among adults admitted to a small local detention center, there is a wealth of empirical evidence available to support the connections between SUDs and criminal justice contact. The most relevant recent work in this area has found self-reported drug use to be associated with higher readmission counts among pretrial detainees in New York City (Kim et al., 2018). Although the results from the current study add key information to this discussion, additional research with more diverse samples of adults in local correctional centers may examine the relationships between SUDs for specific substances and the likelihood of jail readmission for certain offenses to further refine the knowledge in this area.

These findings also emphasize the importance of the collection of behavioral health data for many criminal justice practitioners, including jail administrators and correctional officers, who are responsible for the safety of personnel and detainees within these facilities. Research has shown this information can be used by correctional staff to identify those who may be at greater risk for misconduct within the facility (Houser et al., 2012; Houser & Belenko, 2015; Wood, 2014). It is also well-known that many jails do not have the staff to address these needs, which makes collecting this information that much more difficult, but also that much more important. Understanding the needs of adults in the facility can help justify the need for additional personnel within the facility while giving jails with the resources to triage and address these conditions the ability to provide targeted interventions or plan to deliver services to inmates in need. All of these options require varying levels of budgetary resources, but the common thread is that they are all tied back to the systematic collection and utilization of detailed behavioral health indicators.

Outside of the facility, these results contribute to the growing body of evidence demonstrating SUDs may be more potent indicators of the probability of jail readmission relative to MHCs (Bonta et al., 2014; Ferguson et al., 2009; Wilson et al., 2011). This should not be interpreted as an indication that MHCs can be overlooked, but instead that the focus should be concentrated on the identification of SUDs as early as possible to avoid continual reliance on the local jail as the primary method of managing adults with these conditions.

The sequential intercept model proposes that the earlier that these conditions are identified, the more immediate the mounting of a clinical response (Munetz & Griffin, 2006). This approach is designed to link adults with requisite services with the objective of reducing reliance on the local jail as the primary method to manage behavioral health needs among adults. Amphetamine and opiate use disorders are currently the primary concerns for this jail population and the surrounding community, stressing the need to initiate specific treatment oriented-approaches within the facility with the goal of connecting adults to community-based programming upon release. Short of addressing these needs, adults with SUDs will continue to be cycled in-and-out of this jail and it is likely that many other small-to-medium sized facilities experience the same problems.

The facility in which this study was conducted has used this information to implement new practices aimed at identifying behavioral health needs of adults prior to multiple jail readmissions. One of the newly adopted methods involves the addition of a clinical team who meets with recently admitted inmates to ascertain detainees’ behavioral health needs. The primary functions of this team is to facilitate a warm hand-off to a community-based program immediately upon release for adults in need. This program has conveniently relocated adjacent to the detention center to better serve adults during their transition back into the community.

Collaborative efforts between the sheriff’s office and the local police department have also been developed to identify adults with severe opiate use disorders with the aim of diverting them from the jail toward appropriate treatment services. This initiative remains in the early stages of development, but it will operate according to the primary goal of the intercept model by identifying adults with treatment needs at the earliest possible point in the criminal justice system. Law enforcement will be given the ability to provide an alternative to arrest by referring eligible adults to treatment rather than booking them into the jail.

The current study provides valuable insight into the characteristics associated with multiple jail readmissions, but there are some limitations that must be considered. Although the study site may be representative of smaller correctional facilities in rural areas, the sample was limited in its racial and ethnic diversity, which requires a note of caution in generalizing these findings to other areas containing greater racial and ethnic minority group representation. The prospective research design of this study offers some strength in establishing a temporal relationship between the presence of SUDs, MHCs, and the likelihood of readmission into the correctional facility, but it did not collect information related to the precise onset of behavioral health conditions or exact measures of how long participants had been experiencing certain symptoms. Thus, the study was unable to ascertain the extent to which the jail experience triggered or possibly exacerbated certain conditions. Lastly, readmission data were collected strictly from the correctional facility that served as the study site. Data related to readmissions into facilities in surrounding areas was not available for the current study, and it is possible that some detainees were booked into other facilities during the 12-month follow-up period and these jail bookings went unrecorded.

## Conclusion

Of the most prevalent behavioral health indicators observed among adult jail inmates, moderate-to-severe SUDs were the most robust predictors of repeated admissions. This is especially problematic considering estimates that suggest more than two thirds of the jail population who met criteria for SUDs or MHCs did not receive any treatment or counseling service while incarcerated (Sung et al., 2010). This means the majority of adults who enter jails do not actually receive any services to address one of the most prominent reasons related to why they were admitted to the facility in the first place. Despite the established rights to physical and mental health care (Klein, 1979), in most instances it is likely that these conditions are not even identified. Under these circumstances, it should come as no surprise that readmission rates are so high for adults with moderate-to-severe SUDs, but optimistically speaking, jail booking can be conceived as an opportunistic intervention point. Collecting information related to SUDs and MHCs serves as an initial step in the process to begin to address some of the underlying reasons related to jail admission, and this can also contribute to the development of specialized community programs to divert adults with these needs out of jails altogether.
